# Ivabradine in Postural Orthostatic Tachycardia Syndrome: A Review of the Literature

**DOI:** 10.7759/cureus.7868

**Published:** 2020-04-28

**Authors:** Faryal Tahir, Taha Bin Arif, Zainab Majid, Jawad Ahmed, Muhammad Khalid

**Affiliations:** 1 Internal Medicine, Dow University of Health Sciences, Karachi, PAK; 2 Cardiology, Kansas City University of Medicine and Biosciences, Joplin, USA; 3 Cardiology, Ascension Via Christi Hospital, Pittsburg, USA

**Keywords:** postural orthostatic tachycardia syndrome, postural tachycardia syndrome, pots, ivabradine, neuropathic pots, hyperadrenergic pots, hypovolemic pots, autoimmune pots, deconditioning pots, heart rate-lowering drug

## Abstract

Postural orthostatic tachycardia syndrome (POTS) is an autonomic disorder characterized by symptoms such as palpitations, dyspnea, chest discomfort, and lightheadedness affecting various systems. The pathophysiology of POTS is not completely understood due to a variety of symptoms showing that the disease is multifactorial. There is no approved uniform management strategy for POTS and hence, no drug has been approved by the United States (US) Food and Drug Administration (FDA) for it. Ivabradine is an FDA-approved drug for stable symptomatic heart failure (HF) and patients with an ejection fraction (EF) of ≤35%. Previous studies have depicted improvement in symptoms of POTS with the use of ivabradine. It is a selective inhibitor of funny sodium channels (I_f_) in the sinoatrial (SA) node cells resulting in the prolongation of the slow diastolic depolarization (phase IV) and reduction in the heart rate (HR). Although beta-adrenoceptor blockers are commonly used to lower HR in patients with POTS, they are less ideal due to numerous adverse effects. This review aims to provide a comprehensive and up-to-date picture of all the studies and case reports that utilized ivabradine for the treatment of POTS along with a precise overview of epidemiology, pathophysiology, and types of POTS. To conclude, we recommend further research on the effectiveness of ivabradine in patients who experience symptoms of POTS. Other than stable chronic angina pectoris, its application in this setting has been proven to be effective and safe. Further evaluation by means of randomized control trials is required to encourage use of this HR-lowering agent in common disorders other than HF and stable angina, i.e. POTS.

## Introduction and background

The first informal mention of postural orthostatic tachycardia syndrome (POTS) was by Da Costa, in 1871, who referred to it as “soldier’s heart” or “irritable heart” [1]. However, Schondorf and Low, in 1993, first described POTS in the adult population as an increase in the heart rate (HR) in a symptomatic patient by more than 30 beats per min (bpm) when the patient moves from supine to upright position [2]. In 2015, Heart Rhythm Society defined POTS on the basis of three points: (1) a clinical syndrome characterized by symptoms of lightheadedness, blurring of vision, palpitations, intolerance to exercise, and fatigue; (2) an increase of ≥30 bpm (≥40 bpm in those aged 12-19 years) in the HR when the person stands up from a recumbent position; and (3) absence of orthostatic hypotension [3]. Orthostatic hypotension is characterized by a more than 20 mmHg drop in systolic blood pressure (BP) on standing [3]. The incidence of POTS varies globally from 0.2% to 1% in the developed countries with an increased prevalence among females, Caucasian race, and individuals from 13 to 50 years of age [4,5,6-8]. The affected individuals account for 3,000,000 cases alone in the United States of America (USA) [9]. A recent 2019 study has shown that the incidence of POTS has increased fourfold since 2000 [8].

POTS is an autonomic disorder characterized by symptoms such as palpitations, dyspnea, chest discomfort, lightheadedness, nausea, blurred vision, chronic fatigue, sleeping abnormalities, migraines, hypermobile joints, abdominal pain, irritable bowel, and bladder symptoms as well affecting various systems [9,10]. Only 30% of individuals have reported fainting along with the symptoms of POTS [9]. Usually, there is a two-year (median) delay in the diagnosis of disease from the onset of symptoms [7]. The pathophysiology of POTS is not completely understood due to a variety of symptoms showing that the disease is multifactorial [4,9,10]. Chronic fatigue syndrome, inappropriate sinus tachycardia, and vasovagal syncope are few conditions associated with POTS [4].

There is no approved uniform management strategy for POTS and hence, no drug has been approved by the US Food and Drug Administration (FDA) for it [4]. Non-pharmacological therapies include lifestyle modifications such as increased hydration and salt intake, and use of support stockings [11]. Pharmacological therapies include beta-blockers (first line), alpha-agonists (first or second line), mineralocorticoids (second line), selective serotonin reuptake inhibitors (SSRIs), and selective serotonin-norepinephrine reuptake inhibitors (SSNRIs), and rarely used drugs include pyridostigmine, desmopressin, and erythropoietin [4,11]. However, there has been evidence of beneficial outcomes with the use of ivabradine in POTS patients, as seen in prospective and retrospective studies [12-16].

Ivabradine is an FDA-approved drug for stable symptomatic heart failure (HF) and patients with an ejection fraction (EF) of ≤35% [17,18]. European Society of Cardiology recommends ivabradine as second-line therapy for patients whose angina has been poorly controlled by other medications, namely calcium-channel blockers (CCBs), beta-blockers, or nitrates (short-acting) [19].

Ivabradine increases the diastolic time and reduces the HR by inhibiting channels responsible for maintaining cardiac pacemaker current, I_f_ (funny current). The selective blocking of these trans-membrane ion channels that conduct the inward depolarizing sodium (Na) and potassium (K) current slows down the HR without affecting systemic vascular resistance and cardiac inotropy [18,20,21]. Ivabradine has been associated with many severe side effects such as bradycardia, heart block, sinus arrest, QT prolongation, torsades de pointes, and fetal toxicity. Other less severe side effects include, but are not limited to, vertigo, diplopia, rash, and hypotension [17,18].

Ivabradine is not an FDA-approved drug for POTS but due to its ability to reduce HR, it has shown improvement in POTS patients in many studies [12-16]. This review aims to provide a comprehensive and up-to-date picture of all the studies and case reports that utilized ivabradine for the treatment of POTS along with a precise overview of epidemiology, pathophysiology, and types of POTS.

## Review

**Figure 1:**
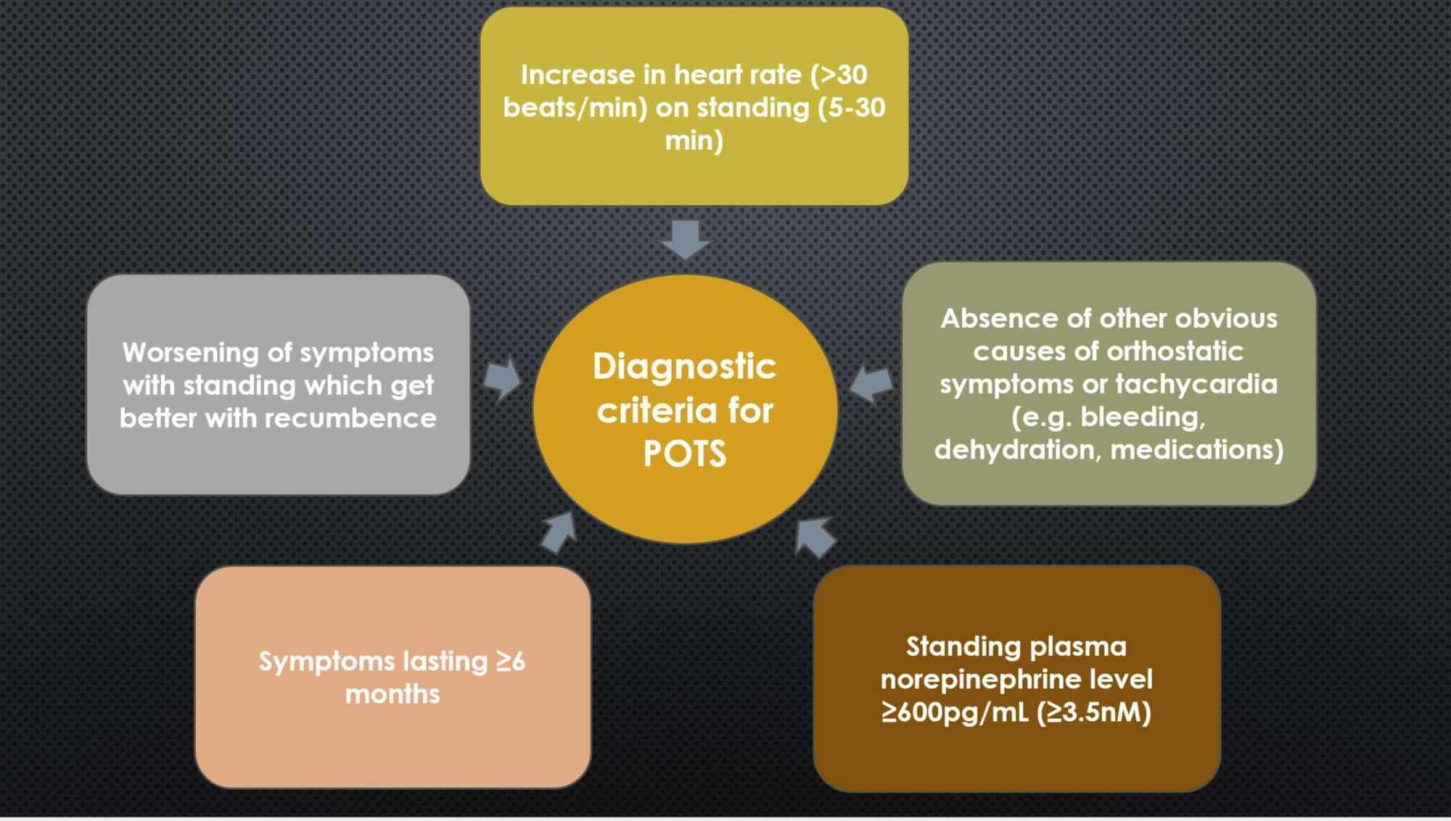
Diagnostic criteria for POTS Adapted from [10]. POTS: postural orthostatic tachycardia syndrome

POTS is a heterogeneous blood circulation disorder characterized by orthostatic hypotension and tachycardia with the HR increasing by at least 30 bpm in adults or by 40 bpm in adolescents during the first 10 minutes of standing from a recumbent position [3,11]. Patients with POTS often complain of postural tachycardia, headache, abdominal discomfort, dizziness, pre-syncope or syncope, nausea, tremors, anxiety, sleep disturbance, and chronic fatigue that significantly impair their quality of life [11]. The diagnostic criteria for POTS are illustrated in Figure 1.

Previously, POTS was labeled as “soldier’s heart” or “irritable heart” when it was first reported, in 1871, by Da Costa who described inappropriate tachycardia in civil war soldiers [1]. However, it has also been misdiagnosed as psychiatric illness such as depression and anxiety in recent times [10].

Prevalence

Being classified as the most common type of orthostatic intolerance (OI), POTS affects about 1% of the US population [2,9]. A majority of patients diagnosed with POTS fall in the 13- to 50-year age group [6]. Women are found to be more afflicted by POTS with a ratio of 4-5:1 [22]. A retrospective study conducted in the pediatric population of Philadelphia reported the demographics of POTS that showed a higher incidence among Caucasians (94.1%) and females (77.5%). Furthermore, it was noticed that the median age of onset of symptoms in patients under 18 years of age was 13.1 years with a median duration of 2 years before diagnosis [7].

Etiology and types of POTS

The etiology of POTS varies with its types, as described below in Table 1, resulting in tachycardia secondary to cardiovascular deconditioning. 

**Table 1:** Types of POTS with description POTS: postural orthostatic tachycardia syndrome, NE: norepinephrine, NET: norepinephrine transport, HR: heart rate, TCAs: tricyclic antidepressants, RBCs: red blood cells, RAAS: renin-angiotensin-aldosterone system, RA: rheumatoid arthritis, SLE: systemic lupus erythromatosus, ANAs: antinuclear antibodies

Type of POTS	Description
Neuropathic POTS	Neuropathic POTS is an autonomic neuropathy characterized predominantly by lower limb sympathetic denervation leading to reduced venoconstriction and venous pooling [23]. One study highlighted that despite normal NE spillover, POTS patients exhibited decreased NE release in the lower extremities demonstrating dysfunctional NE reuptake secondary to injured peripheral nerves [24].
Hyperadrenergic POTS	Approximately 30%-60% of POTS patients fall under the category of hyperadrenergic type. An elevated standing plasma NE level of ≥600 pg/mL with an increased sympathetic tone manifesting as palpitations, tremors, hypertension, tachycardia, and anxiety is characteristic [23]. One case depicted the loss-of-function mutation of the SLC6A2 gene resulting in deficient NET and elevated mean supine HR [25]. Medications like TCAs, sympathomimetics (e.g., methylphenidate), and NE reuptake inhibitors (e.g., bupropion) are more frequently seen to cause NET block [23].
Hypovolemic POTS	Although the severity of hypovolemia varies between studies, about 70% of patients with POTS show decreased plasma, RBCs, and total blood volumes [3,23,26]. An impairment in the RAAS associated with a lower level of renin and aldosterone can lead to low volume state in POTS patients [27].
Autoimmune POTS	Considering the similarities (female predominance, post-viral onset, elevated autoimmune markers) with other autoimmune disorders like RA, SLE, and sjogren syndrome, an autoimmune hypothesis has been proposed for POTS [28]. POTS patients have been observed to produce increased autoantibodies. A study found that 25% of patients had positive ANAs with Hashimoto’s thyroiditis as the most prevalent disorder in these individuals [29].
Deconditioning POTS	POTS patients often have physical and cardiovascular deconditioning, but its presence as either cause or effect is unidentified. Furthermore, the degree of deconditioning does not inevitably correspond to objective laboratory and autonomic findings [3].

Pathophysiology of POTS

POTS has heterogeneous pathophysiology, including excessive sympathetic stimulation, defective peripheral autonomic function, hypovolemia, or autoimmune dysfunction [4,23,28]. Orthostatic pathologies reflect gravity-dependent physiology as the total effective circulatory volume reduces due to the shift of intravascular volume to interstitial space. As a consequence, an increase in the sympathetic stimulation manifested as increased cardiac contractility, HR, and peripheral vascular resistance (PVR) occurs secondary to a subsequent reduction in stroke volume. Despite an extravagant sympathetic response to postural changes such as standing, POTS patients present with persistent decreased volume, consummating into a common terminal pathway of tachycardia in the presence of OI on standing [28]. However, the mechanism behind the pathophysiology of POTS is not completely understood due to the complex overlap of cardiovascular, neuropathic, renal, immune, and hematologic systems [5].

Management of POTS

POTS is treated by both pharmacological and non-pharmacological approaches that solely depend on the definitive diagnosis, patient education, and compliance with the therapy.

Due to its non-specific and chronic debilitating nature, the management of POTS relies on the patient’s knowledge and management of expectations. The need for a multi-faceted therapeutic approach through the assessment of patient’s understanding and follow-ups on questions and concerns cannot be overemphasized [5]. Regardless, each approach should be customized according to the predominant subtype (e.g., hypovolemic, hyperadrenergic).

The primary aspect of POTS treatment is exercise conditioning and all patients are recommended to start a gradual physical exercise regimen [5]. Considering that the symptoms may exacerbate with increased activity, the patient can begin with low-intensity exercise programs concentrated on avoiding upright posturing (e.g., swimming) with incremental progression over three months [4,28]. A study found that 10 out of 19 (53%) patients no longer met POTS criteria following a three-month exercise regimen. Furthermore, an 11% increase in maximal oxygen intake, 12% increase in left ventricular mass, and 8% rise in end-diastolic volume were noticed in these patients [30]. Other non-pharmacologic interventions include increased fluid and salt intake, compression garments, and physical counter-maneuvers like leg crossing, muscle contraction, and forward bending [23,31].

Pharmacologic therapy is not considered a first-line intervention for POTS and is usually administered in severe or refractory cases [4]. There is no proven clinical effectiveness of pharmacologic therapies over conservative non-pharmacologic interventions, although the patient should be monitored for potential adverse effects (AEs) and drug interactions. There is no approved medication for the treatment of POTS by the US FDA [4,28]. Some common pharmacologic agents that are used off-label for the treatment of POTS are summarized below in Table 2.

**Table 2:** Pharmacologic agents for the treatment of POTS HF: heart failure, POTS: postural orthostatic tachycardia syndrome, PVR: peripheral vascular resistance, BP: blood pressure, HR: heart rate

Drug	Features
Fludrocortisone	It is a synthetic mineralocorticoid that promotes renal Na reabsorption and loss of K. Adverse effects include hypertension, hypokalemia, headache, edema, and HF [28].
Midodrine	It is an alpha-1 adrenergic agonist that increases arteriolar and venous tone resulting in an increased venous return that can be beneficial for hypovolemic phenotypes. Supine hypertension, paresthesias, urinary retention, and urgency are some of the limitations [3].
Clonidine and alpha-methyldopa	Hyperadrenergic POTS patients with hypertension can benefit from these centrally acting alpha-2 agonists. They reduce sympathetic tone and decrease PVR, BP, and HR. However, the patient should be monitored for cognitive clouding, headache, skin rash, fatigue, and sedation [28].
Propranolol, metoprolol	Beta-adrenergic blockers decrease HR and cardiac contractility by blocking beta-adrenergic receptor activation. Non-selective beta-blockers (propranolol) can also reduce splenic vasoconstriction via beta-2 inhibition. Bradycardia, hypotension, fatigue, and syncope are common adverse effects [28].
Pyridostigmine	It is an acetylcholinesterase inhibitor that increases acetylcholine levels in autonomic ganglia and peripheral muscarinic receptors. its continual use is limited by abdominal pain, diarrhea, vomiting, and dysmenorrhea [4].

Medications that aggravate specific symptoms like tachycardia (e.g., sympathomimetics including amphetamines, SSRIs, and SSNRIs) or deteriorate OI (e.g., CCBs, diuretics, opiates, nitrates, and tricyclic antidepressants) should be avoided [28].

Ivabradine for the treatment of POTS

**Figure 2:**
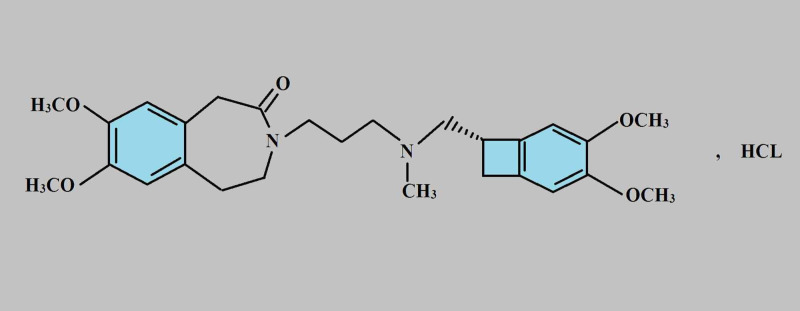
Structure of ivabradine Adapted from [20].

Ivabradine is a selective inhibitor of funny sodium channels (I_f_) in the sinoatrial (SA) node cells resulting into the prolongation of the slow diastolic depolarization (phase IV) and eventually, reduction in HR [32]. In 2015, FDA approved ivabradine for reducing the risk of hospitalization for worsening HF in adults with stable, symptomatic chronic HF with reduced EF and for the treatment of stable, symptomatic HF due to dilated cardiomyopathy in pediatric patients (aged 6 months or older) [20]. Ivabradine can also be used for patients with chronic stable angina who cannot take beta-blockers. It reduces HR by decreasing myocardial oxygen demand and maintaining the viability of myocardial cells. In addition, it prolongs diastolic perfusion time and improves coronary flow velocity reserves. These physiological changes result into an increased ischemic threshold and an improvement in angina [33]. Ivabradine reduces HR almost linearly with an oral dose ranging from 0.5 to 24 mg. However, HR is reduced nonlinearly reaching a plateau at higher doses [34]. Ivabradine has no significant effect on BP as it does not affect myocardial contractility [35]. It has minimal AEs and a relatively better tolerance according to previous clinical trials. Some common AEs include bradycardia, hypertension, atrial fibrillation (AF), luminous phenomena, or visual brightness [20].The molecular structure of ivabradine is shown in Figure 2 [20].

Beta-adrenoceptor blockers are commonly used to lower HR in patients with POTS; however, they are less ideal due to numerous AEs. Although not FDA approved, previous studies have depicted improvement in symptoms of POTS with the use of ivabradine. According to a study by Ruzieh et al., the best response rate (88.4%) was shown for palpitations, a considerably troubling and the most prevalent symptom among these patients [16]. The improvement is highly linked to the nature of ivabradine as a selective SA node blocker that decreases HR without affecting cardiovascular physiology. The response to ivabradine was higher in their cohort as compared to previous studies with a significant improvement in other symptoms, such as lightheadedness (76.1%), syncope (60.7%), fatigue (57.5%), trouble concentrating (55.2%), and dyspnea (42.9%) [16]. Similarly, studies by Sutton et al. and McDonald et al. demonstrated that ivabradine may be beneficial in conditions where sinus tachycardia is the main feature [12,14]. Ivabradine showed improvement in 18 out of 25 patients with vasovagal syncope and tachycardia on the tilt table test with no major AEs [12].

POTS and ivabradine: what do studies say?

A total of five researches were identified that studied the use of ivabradine in POTS patients either prospectively or retrospectively.

Sutton et al. published a pilot study, in the year 2014, in which the effect of ivabradine was assessed in patients with vasovagal shock who manifested with sinus tachycardia on the head tilt, meeting the HR criteria for POTS [12]. The study population was 25 patients, with a mean age of 33 years, who presented with syncope, 92% of whom also experienced palpitations, during the period of 2008 to 2011. Ivabradine with a standard dose of 5 mg/day was initiated, with a maximum dose of 20 mg/day. The resting HR was charted that showed no signs of symptomatic bradycardia; however, repeat tilt testing was not performed. The drug was continued by all patients for a duration of 2-40 months with a mean dosage of 10.7 mg/day as once or twice daily medication. The results showed a complete resolution of symptoms in 32% of the patients, whereas 40% experienced great improvement in syncope. Remaining 28% of the patients discontinued the drug due to no improvement of symptoms, pregnancy, or side effects, the latter been reported as either non-specific or related to the retina [12].

In 2015, Barzilai and Jacob studied the effects of ivabradine on HR and BP in eight patients suffering from POTS for the past two years [13]. Following a series of the experimental protocol, the patients initially had their BP, HR, and RR measured and were laid supine on a tilt table for 30 minutes. This was followed by a sampling of the previously mentioned parameters and evaluation of the sympathetic and parasympathetic systems. The patients were then head uptilted at 70° for 20 minutes and finally rested in the supine position. Ivabradine was administered at a single dose of 7.5 mg, along with the repetition of the protocol after 60-80 minutes. The drug was found effective in reducing the resting HR by 4 ± 1 bpm with a notable decrement from 118 ± 4 bpm to 101 ± 5 bpm after five minutes of head tilt. Moreover, the drug did not alter the BP and cardiovascular vagal and sympathetic tone and showed no significant side effects [13].

Another research was carried out, in the UK, by McDonald et al. analyzing similar effects of the drug on patients with POTS [14]. This retrospective case study was carried out on 20 patients from a tertiary cardiovascular ward who participated by filling a self-assessment tool, discussing their perception of drug effectiveness, changes in symptoms, and side effects. Nearly half (55%) of the patients continued the use of ivabradine at the time of analysis with a mean duration of 25 weeks and mean drug dosage of 5 mg in a single dose or two doses, and reported decreased episodes of palpitations and tachycardia. Improvement in fatigue was reported by 44% of the participants. Side effects were experienced by five patients, two of whom complained of visual disturbances and increased fatigue, the patient with the latter terminating his treatment [14].

Through the period of February 2008 to June 2014, Donne et al. retrospectively evaluated the pediatric population, less than 18 years of age, with POTS for the effects of ivabradine [15]. Using the pharmacy database, 22 patients were prescribed an initial dose of 5 mg/day in two divided doses, and in 50% of the patients, the dose was adjusted with the mean value being 9.5 mg. During the median follow-up of 4.6 months, the majority (68%) of the participants reported improved symptoms and decreased episodes of syncope. However, in six patients, the follow-up was of less than three months; ivabradine was terminated in four of them accounting for resolution of symptoms (n=2), no improvement (n=1), or worsening of syncope and palpitation (n=1). Side effect of mild phosphine was observed in only one patient. Decrement in the HR on resting electrocardiogram (EKG) analysis was observed from a mean of 82.5 to 71, performed in 86% of the patients. Overall, the drug was considered safe for use in children less than 18 years of age [15].

A recent study carried out, in 2017, by Ruzieh et al. determined the efficacy of ivabradine in a comparatively larger sample size of 49 patients, the majority being females [16]. Based on the more substantial part of the cohort (78%) reporting a noteworthy improvement, this study, like the others before, also concluded on the drug being productive for the treatment of POTS, given further placebo-controlled trials should be carried out. The initial dose of ivabradine in this study was 2.5 mg in two doses and was tittered according to each patient. Side effects observed were related to visual brightness in nine and nausea in four patients. In follow-up values of vitals, no change in BP was recorded, whereas HR in sitting and standing positions was found to be lowered [16].

Table 3 summarizes all the above-mentioned studies evaluating outcomes of ivabradine therapy among POTS patients.

**Table 3:** Summary of studies evaluating outcomes of ivabradine in POTS VVS: vasovagal syncope, POTS: postural orthostatic tachycardia syndrome, HR: heart rate, bpm: beats per minute

Author (year)	Study type	Study duration	Objective	No. of patients (mean age)	Drug (dosage)	Mean duration of treatment	Results	Adverse reactions
Sutton et al. (2014) [12]	Prospective open-label trial	October 2008-December 2011	To evaluate the response of a subgroup of VVS patients to ivabradine.	25 (33 years)	Ivabradine (10.7 mg/day)	15 months	72% of the patients gained benefit from ivabradine with 32% becoming completely asymptomatic.	Visual side effects (9%), unspecified side effect (4%)
Barzilai et al. (2015) [13]	Prospective open-label trial	-	To study the effect of Ivabradine on the hemodynamics and sympathovagal balance in POTS patients.	8 (31 ± 3 years)	Ivabradine (7.5 mg)	Single dose	Ivabradine decreased resting HR by 4 ± 1 bpm and from 118 ± 4 to 101 ± 5 bpm after tilting for five minutes.	-
McDonald et al. (2011) [14]	Retrospective case-series	January 2008-July 2010	To evaluate POTS patients treated with ivabradine.	20 (35 ± 9.9 years)	Ivabradine (5 mg/day)	25 weeks	Among patients treated with ivabradine, 60% report a symptomatic improvement.	Visual abnormalities (10%), dizziness (5%), fatigue (5%)
Delle Donne et al. (2017) [15]	Retrospective cohort study	February 2008-June 2014	To review the experience of ivabradine evaluation among pediatric patients with POTS.	22 (14.5 years)	Ivabradine (9.5 mg/day)	3.7 months	Among POTS patients younger than 18 years of age, ivabradine produced improvement of symptoms in 68%.	Mild phosphenes (4.5%)
Ruzieh et al. (2017) [16]	Retrospective cohort study	January 2010-October 2016	To examine the effects ivabradine among POTS patients.	49 (35.1 ± 10.35 years)	Ivabradine (10.9 mg/day)	3-12 months	Showing the efficacy of ivabradine in POTS patients, nearly 78% of the cohort reported a significant improvement in symptoms.	Visual brightness (18%), nausea (8.2%)

POTS and ivabradine: a review of case reports

To further expand our knowledge regarding the practical implementations of ivabradine, we have taken the support of previously published case reports and analyzed the outcomes in terms of its effectiveness and side effects along with treatment duration (Table 4). All case reports published till date that discussed the outcome of ivabradine in POTS patients of every age and race were taken for the evaluation. An evaluation of these case reports shows 88% (eight out of nine) cases with female patients, and this high female-to-male ratio is consistent with the previously published literature (Table 4) [12-14,16]. The mean age of the patients with symptoms of POTS and sympathetic dysfunction has been found to be 26.8 years, ranging from 17 to 42 years [36-44]. This figure is also coherent with the past studies of ivabradine assessment, in which the affected individuals were young and middle-aged adults. The patients with POTS usually experience symptoms of fluctuating HR and BP, lightheadedness on standing, extreme fatigue, nausea, and anxiety among other orthostatic symptoms. Patients among reported cases mostly experienced fatigue and palpitations, syncope, and lightheadedness on standing. Veins in the lower legs may particularly be affected by the sympathetic denervation, causing them to become atonic and giving rise to subsequent pooling of blood, and hence, the bluish discoloration of limbs experienced by some individuals [13]. Oztunc et al. described the symptoms of bruising, redness, and swelling of the hands and feet along with dizziness and tachycardia, the former probably accounting for the coexistence of Raynaud's phenomenon [36].

**Table 4:** Summary of case reports assessing effects of ivabradine in POTS HR: heart rate, bpm: beats per minute, BP: blood pressure, TD: three times a day, FIS: Fatigue Impact Scale, OGS: Orthostatic Grading Scale, CHB: complete heart block, PAF: paroxysmal atrial fibrillation, AV: atrioventricular, CSM: carotid sinus massage, AF: atrial fibrillation, LOC: loss of consciousness, DM: diabetes mellitus, POTS: postural orthostatic tachycardia syndrome

Author (year)	Patient age (in years), sex	Presenting symptom	Ivabradine dose	Treatment period	Outcome of treatment	Adverse effects	Other information
Oztunc et al. (2016) [36]	17, female	Bruising, redness, and swelling on the hands and feet after moving from supine to upright position	-	6 months	No complaint of dizziness, bruising, or palpitations during follow-up	-	Tilt table test increased HR (from 80 to 128 bpm) and BP (from 100/60 to 130/90 mmHg); metoprolol 1 mg/kg/day (2 months) and midodrine 10 mg TD (45 days) were given that showed no beneficial effect
Ewan et al. (2007) [37]	21, female	Fatigue (FIS=102), orthostatic intolerance (OGS=19), palpitations (HR=120-160)	2.5 mg 12-hourly, then 5 mg 12-hourly	-	Improvement in fatigue (FIS=52), orthostatic intolerance (OGS=9), palpitations (HR=90-95)	-	Patient was unable to tolerate beta-blockers and verapamil due to asthma and nausea, respectively.
Khan et al. (2009) [38]	44, female	Palpitations on standing (107-140 bpm)	5 mg 12-hourly	6 weeks	Reduction of supine and erect HR to 80 and 90 bpm, respectively, HR= <120 bpm at 6-week checkup	Mild transient visual disturbances (treatment continued despite adverse effect)	Dual-chamber pacemaker implanted for intermittent CHB, history of PAF, beta-blockers poorly tolerated (Raynaud’s syndrome)
Jamil-Copley et al. (2010) [39]	25, female	Recurrent pre-syncopal and syncopal episodes (HR=100 bpm and BP=140/80 mmHg while seated and BP=160/90 mmHg on standing)	5 mg 12-hourly	3 weeks	Dissolution of palpitations (HR=90 bpm) and syncopal episodes	-	Patient was a known case of essential hypertension (took ramipril and bisoprolol); tilt table test produced sinus tachycardia (HR=146 bpm)
Nakatani et al. (2011) [40]	42, female	Postural palpitations and syncope	2.5 mg/day	Ivabradine was discontinued after adverse reaction	Ivabradine effectively reduced resting and standing HR, improved orthostatic symptoms with no recurrence of syncope	Allergic reaction	Head-up tilt test increased HR to 120 bpm without hypotension; alpha-agonists, beta-blockers, Ca^2+^-channel blockers, and digitalis were not tolerated; sinus and AV node ablation with pacemaker implantation was performed after terminating ivabradine
Aliyev et al. (2010) [41]	30, male	Frequent syncopal attacks precipitated during upright position and CSM, PAF	5 mg 12-hourly	5 days (short-term therapy) and 6 months (follow-up)	Short-term therapy resulted in disappearance of syncopal episodes during upright position and CSM; no syncope or AF on follow-up	-	Syncopal attacks associated with HR=140 bpm plus sudden drop in BP
Cheema et al. (2019) [42]	19, female	Postural syncopal episodes preceded by palpitations	2.5 mg 12-hourly	6 months	Ivabradine resulted in improvement of symptoms and negative repeat tilt tests	-	The patient took excessive hydration, salt, and fludrocortisone with no relief; patient had a history of head concussion 3 years ago; tilt table test increased the HR up to 148 bpm followed by LOC
Hersi (2010) [43]	25, female	Fatigue, severe weakness and palpitations on standing (HR=139 bpm, BP=125/70 mmHg), tingling and coldness in feet	5 mg 12-hourly	2 days (short-term therapy) and 4 months	Short-term therapy made her able to stand without weakness or tachycardia (HR=80 bpm)	-	Patient ran out of Ivabradine after 4 months, symptoms recurred, then resolved again upon reinstituting the drug
Meyer et al. (2015) [44]	19, female	Postural palpitations (HR=121-131 bpm on standing) and lightheadedness	-	3 months	Ivabradine alleviated resting tachycardia, postural palpitations, and lightheadedness	-	POTS followed a stressful event; patient was a known case of DM type 1; psychosocial support was given

Before the administration of ivabradine, some patients had gone through other pharmacological treatment modalities for the relief of symptoms. In majority of the cases, the patients had a history of using beta-blockers while a few also reported alpha-blockers and CCBs [36-41]. In some cases, beta-blockers were discontinued due to poor tolerance whereas Oztunc et al. reported changing the treatment option to ivabradine due to no effective outcome from metoprolol and midodrine [36-38,40]. Another patient complied with the advice of maintaining hydration and taking fludrocortisone for several months only for her symptoms to persist [42].

The dosage of ivabradine in the mentioned nine cases varied from 2.5 to 5 mg 12-hourly, and based on individual assessment of the patients, the dose was either tittered, maintained, or terminated (Table 4). The duration of treatment with ivabradine was variable with the earliest response observed just after two days of administration in a 25-year-old-female who had presented with symptoms for the past two weeks [43]. The patient did not experience symptoms of weakness or tachycardia on standing along with improvement in standing HR from 139 to 80 bpm. In other case reports, a complete resolution of syncopal episodes was also observed during the course of follow-up while the patient remained asymptomatic [39,41]. Almost all the other cases reported improvement in standing and supine HRs with a notable recovery from palpitations, tachycardia, and syncope [36,37,42,44].

Although majority of the cases pointed towards the successful and effective outcomes of ivabradine in patients presenting with symptoms of POTS, two cases also brought forward the side effects experienced by patients during the treatment course. Nakatani et al. discussed the management by using ivabradine alongside AV node ablation and pacemaker implantation [40]. Although the drug effectively decreased the orthostatic symptoms, and resting and standing HRs, it, however, had to be discontinued due to some non-specified adverse allergic reaction [40]. Another case was that of a 45-years-old with POTS and dual-chamber pacemaker [38]. A trial with an initial dose of 5 mg in two doses was started and an improvement in supine and erect HRs was observed (from 107-140 to 80-90 bpm); however, mild symptoms of visual disturbances were reported. In light of the improved outcome in her HR and sinus tachycardia, the patient decided to continue with the therapy [38].

In 2018, Gee et al. conducted a meta-analysis and reviewed the available literature to evaluate the efficacy and safety of ivabradine for POTS treatment [45]. The authors included open-label trials and cohort studies with the outcomes of ivabradine in symptomatic POTS. This meta-analysis also evaluated case reports where patients with POTS symptoms were given ivabradine either as a first-line or a second-line drug following failure of beta-blockers to diminish the symptoms. At last, Gee et al. concluded that patients with symptomatic tachycardia, who have experienced failure of treatment with other pharmacologic therapies, can be benefited with a trial of ivabradine. The authors suggested further research to fully illustrate the role of ivabradine for treating POTS patients and to highlight the category of patients more prone to improve their symptoms [45].

## Conclusions

In the light of the previously published literature (retrospective studies, case series, or case reports), we conclude that ivabradine can be safely used in patients with POTS symptoms as very few side effects have been reported along with a dramatic improvement in the symptoms like palpitations, syncope, and falls due to loss of consciousness. Ivabradine can be opted as a second-line treatment in patients with POTS as beta-blockers are mainly used at first. This review highlights that sometimes beta-blockers may fail to resolve the symptoms of POTS. In these scenarios, ivabradine can prove its effectiveness in abolishing the symptoms with uncommon or less severe side effects. To conclude, we recommend further research on the effectiveness of ivabradine that directly and selectively inhibits I_f_ current in the SA node resulting in HR reduction in patients experiencing symptoms of POTS. Other than stable chronic angina pectoris, its application in this setting has been proven to be effective and safe. Further evaluation by means of randomized controls trials is required to encourage the use of this HR-lowering agent in common disorders other than HF and stable angina, i.e. POTS.
